# Supplementary material for: Fumonisin B1 Exposure Causes Intestinal Tissue Damage by Triggering Oxidative Stress Pathways and Inducing Associated CYP Isoenzymes
Source: Toxins (Basel). 2025 May 12;17(5):239. doi: 10.3390/toxins17050239 (PMC12116071; doi:10.3390/toxins17050239)
Supplement: Supplementary file 1 [file toxins-17-00239-s001.zip › toxins-3567621-supplementary.pdf]

Supplementary Table S1. The composition of the basic diet fed to quail was experimentally evaluated for optimal allicin supplementation.

Raw materials	Content (%)
Soybean meal	26.7
Flours	23.8
Maize grain	20.135
Wheat semolina	9.5
Talcum powder	6.5
Fishmeal	4.7
Soybean oil	4
Yeast	1.9
Calcium biphosphate	1.9
Salt	0.5
Choline chloride	0.2
Multi-vitamins	0.08
Composite minerals	0.06
Vitamins	0.025
Add up the total	100

Supplementary Table S2. Nutritional levels of the basal diet fed to quail in the FB_1-allicin diet experiment.

Nutritional levels	Content (%)	Supply ratio (%)
Carbohydrates	52	60
Crude proteins	22	25.5
Fat	5.5	14.5
Crude fibre	3.5	/
Ca	3	/
Total P	0.8	/
